# The has-miR-526b Binding-Site rs8506G>A Polymorphism in the *lincRNA-NR_024015* Exon Identified by GWASs Predispose to Non-Cardia Gastric Cancer Risk

**DOI:** 10.1371/journal.pone.0090008

**Published:** 2014-03-04

**Authors:** Qiu-Hong Fan, Rong Yu, Wei-Xian Huang, Xi-Xi Cui, Bing-Hui Luo, Li-Yuan Zhang

**Affiliations:** 1 Department of Radiotherapy and Oncology, The Second Affiliated Hospital of Soochow University, Suzhou, China; 2 Department of Oncology, Suzhou Municipal Hospital, Affiliated Suzhou Hospital of Nanjing Medical University, Suzhou, China; 3 Department of Gastrointestinal surgery, Wu Jiang first people's hospital, Suzhou, China; MOE Key Laboratory of Environment and Health, School of Public Health, Tongji Medical College, Huazhong University of Science and Technology, China

## Abstract

Gastric cancer including the cardia and non-cardia types is the second frequent cause of cancer-related deaths worldwide. A subset of non-cardia gastric cancer genetic susceptibility loci have been addressed among Asian through genome-wide association studies (GWASs). This study was to evaluate the effects of single nucleotide polymorphisms (SNPs) of long intergenic non-coding RNAs (lincRNAs) on non-cardia gastric cancer susceptibility in Chinese populations. We selected long intergenic noncoding RNAs (lincRNAs) located in non-cardia gastric cancer risk-related loci and identified 10 SNPs located within lincRNA exonic regions. We examined whether genetic polymorphisms in lincRNAs exons are associated with non-cardia gastric cancer risk in 438 non-cardia gastric cancer patients and 727 control subjects in Chinese populations using logistic regression. Functional relevance was further examined by biochemical assays. We found that *lincRNA-NR_024015* rs8506AA carrier was significantly associated with risk of non-cardia gastric cancer (adjusted odds ratio [OR] = 1.56, 95%CI = 1.03–2.39, compared with the rs8506 AG or GG genotype. Further stratification analysis showed that the risk effect was more pronounced in subgroups of smokers (*P* = 0.001). Biochemical analysis demonstrated that the G to A base change at rs8506G>A disrupts the binding site for has-miR-526b, thereby influencing the transcriptional activity of *lincRNA-NR_024015* and affecting cell proliferation. Our present study established a robust association between the rs8506G>A polymorphism in the *lincRNA-NR_024015* exon and the risk of non-cardia gastric cancer.

## Introduction

Gastric cancer (GC) is the second leading cause of cancer death worldwide after lung cancer in 2010, although mortality deaths have decreased slightly from 774,000 in 1990 to about 755,000 in 2010 [1],[2]. Epidemiological studies have showed that environmental factor, including diet, tobacco smoking, alcoholic consumptions and, especially, infection with Helicobacter pylori are associated with a higher risk for GC [3], [4]. Despite of these recognized risk factors, researchers still convinced that genetic factors, particularly single nucleotide polymorphisms (SNPs), are likely to play an essential role in an individual's risk of developing gastric cancer as only a fraction of exposed individuals develop gastric cancer [5].

To date, with the advance of next generation transcriptome sequencing (RNA-Seq), there has been a profound shift in our understanding the entire set of transcriptional aberrations in a disease, including novel transcripts and non-coding RNAs (ncRNAs) not measured by conventional analyses [6]–[9]. Of all of the currently characterized classes of non-coding RNAs molecules, these have been called long intervening ncRNAs (lincRNAs) longer than 200 nucleotides (nt) that are lack an open reading frame and do not overlap protein-coding genes [7], [10]. Groups of lincRNAs have been well characterized to some extent and demonstrated to correlated with important cellular processes such as imprinting, X chromosome inactivation, pluripotency maintenance, and transcriptional regulation [10]–[15]. Furthermore, emerging evidence of dysregulated lincRNA expression in numerous cancers have emerged lincRNA as a new aspect of biology, with evidence suggesting that a major role for involvement of lincRNA in human tumorigenesis and metastasis [16], [17]. Indeed, a well-described example, *HOTAIR* have been studied the contributions to the stepwise progression of tumorigenesis [11], [18], highlighting the role of lncRNAs in cancer biology. In addition, the long noncoding RNA *MALAT1* (metastasis-associated lung adenocarcinoma transcript 1), is frequent misregulation and as a predictive marker for a variety of human cancers of the colon, breast and prostate [19]–[22]. Nevertheless, the mechanisms underlying the specific function of lincRNAs in cancer development has not been fully delineated.

In the past decade, multiple unbiased genome-wide association studies (GWAS) have broadened our understanding of genetic variations related to different types of diseases and cancers by high throughput technologies [23]; however, at least one-third of the identified variants are within non-coding intervals [24]. Recently, two GWAS revealed that several susceptibility risk loci that are associated with non-cardia gastric cancer (NCGC) risk in a Chinese population. Bioinformatics analysis has uncovered numerous lincRNAs close to these loci. Furthermore, several relevant single nucleotide polymorphisms (SNPs) located in the exonic regions of lincRNAs that may associate with NCGC were identified; however, the association between genetic variations in lincRNAs exons and cancer susceptibility has rarely been reported.

In the present study, we hypothesized that SNPs in the exonic region of lincRNAs may altered expression levels and thereby may contribute to NCGC. To test this hypothesis, we conducted a hospital-based case-control study to investigate the associations between these SNPs and susceptibility to NCGC in a Chinese population.

## Materials and Methods

### Study Subjects

All subjects in the current study were ethnically homogenous Han Chinese including 438 NCGC patients and 727 healthy controls. Patients who underwent surgery at the Affiliated Hospitals of Soochow University (Suzhou) were consecutively recruited from 2003 to 2009, with a response rate of 94%. Patients were from Suzhou city and its surrounding regions, and there were no age, sex, and histology restrictions. Details regarding the clinical features of the patients are summarized in **Table 1**. The tumor, node, metastasis (TNM) classification and tumor staging were evaluated according to the 2002 American Joint Committee on Cancer Staging system. Population controls were cancer-free people living in Suzhou region; they were selected from a nutritional survey conducted in the same period as the cases were collected. The control samples were available to us from previous studies which were randomly selected from a database consisting of 3500 individuals based on a physical examination [25]–[27]. The measurement for serum H.pylori immunoglobulin G in NCGC patients and controls was determined by enzyme-linked immunosorbent assay (ELISA). This study was approved by the medical ethics committee of Soochow University. All the participants were genetically-unrelated ethnic Han Chinese and none had blood transfusion in the last 6 months. Having given a written informed consent, each participant was scheduled for an interview with a structured questionnaire to collect selected information, and to donate 5 ml peripheral blood.

**Table 1:** Distributions of select characteristics among non-cardia gastric cancer patients and controls in Chinese populations.

Characteristics	Cases		Controls	
	N (438)	(%)	N (727)	(%)
**Age(years)**				
≤60	156	35.6	283	38.9
>60	282	64.4	444	61.1
**Sex**				
Male	322	73.5	491	67.5
Female	116	26.5	236	32.5
**Smoking status**				
Positive	189	43.2	221	30.4
Negative	249	56.8	506	69.6
**Drinking**				
Positive	175	39.9	267	36.7
Negative	263	60.1	460	63.3
**Family history**				
Positive	34	7.8	51	7.0
Negative	404	92.2	676	93.0
**H.pylori status**				
H. pylori positive	322	73.5		
H. pylori negative	116	26.5		
**TNM stage**				
I	104	23.7		
II	90	20.6		
III	169	38.6		
IV	75	17.1		

### SNP Selection

**Figure 1:**
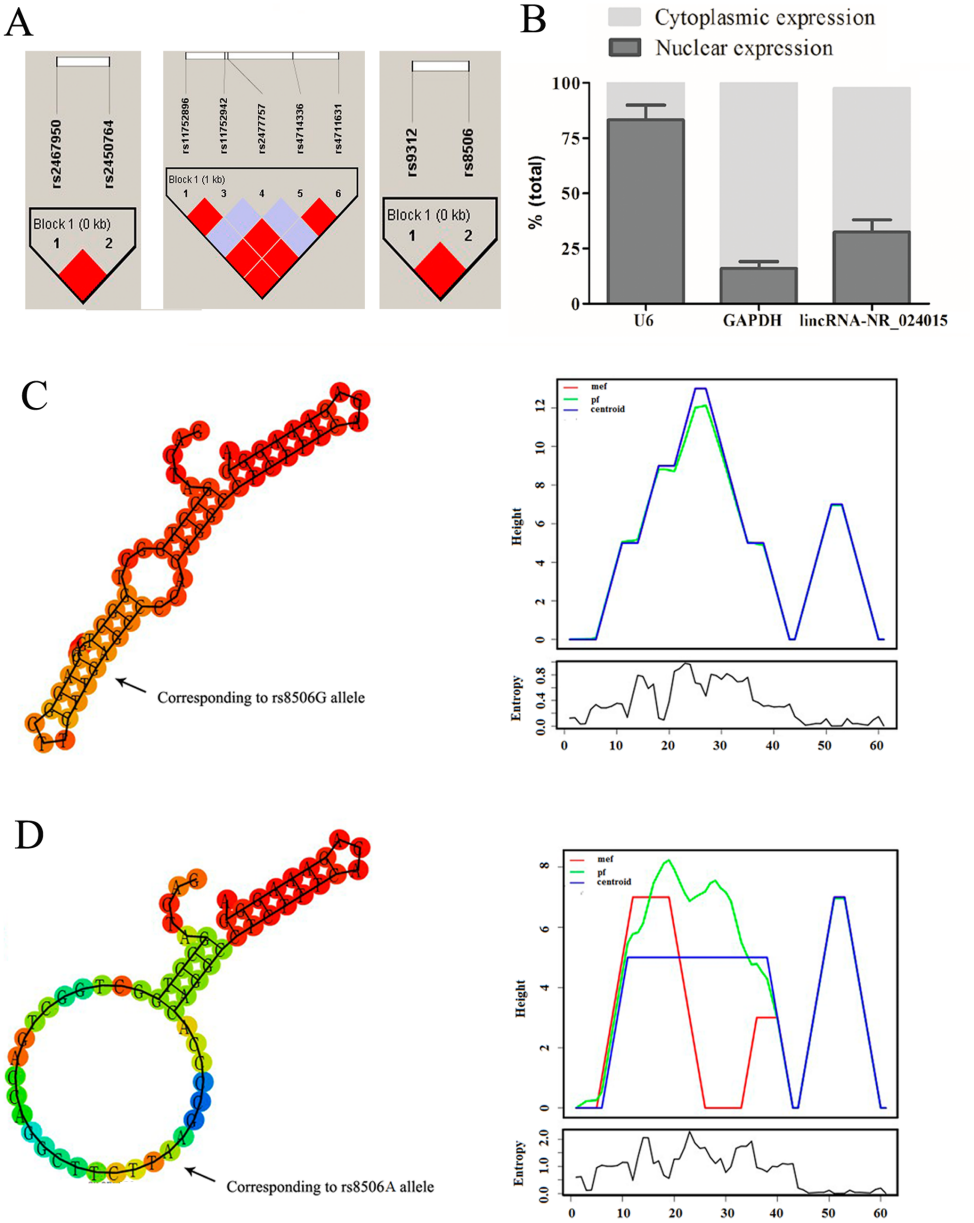
Cellular and molecular characterization of *lincRNA-NR_024015*. (**A**) Linkage disequilibrium (LD) map for selected SNPs of *lincRNA-NR_024015* exon. It showed that rs11752896, rs11752942, rs4714336 and rs4711631 are in LD with D′ = 1; rs2467950 and rs2450764 are in LD with D′ = 1; rs9312 and rs8506 are in LD with D′ = 1. (**B**) The levels of nuclear control transcript (*U6*), cytoplasmic control transcript (*GAPDH* mRNA), and *lincRNA-NR_024015* were assessed by RT-qPCR in nuclear and cytoplasmic fractions. Data are mean±standard error of the mean. Data are presented as a percentage of *U6*, *GAPDH* and *lincRNA-NR_024015* levels and total levels for each were taken to be 100%. (**C–D**) *In-silico* prediction of folding structures induced by rs8506G>A in *lincRNA-NR_024015*. The mountain plot is an xy-graph that represents a secondary structure including MFE structure, the thermodynamic ensemble of RNA structures (pf), and the centroid structure in a plot of height versus position. “mfe” represents minimum free energy structure; “pf” indicates partition function; “centroid” represents the centroid structure.

All published literature investigating an association between genetic susceptibility and NCGC risk were eligible. We searched for studies up to August 2013 using the PubMed database and Web of Science. Relevant search terms were “genome-wide association study”, “GWAS”, “NCGC”, “gastric cancer”, “stomach cancer”, and “Asian”. We also manually searched the reference lists in selected articles. We firstly excluded some articles by scanning the titles and abstracts of studies that were not written in English. Then, after reading the full text of the remaining articles, we identified a final set of studies. All the selected studies met the following criteria: (1) the outcome investigated was based on GWAS in relation to NCGC in humans; (2) the articles were published in English; (3) the latest studies were selected among overlapping data and duplicated data; (4) GWAS was conducted using chip technology. The major exclusion criteria were (1) reviews, tutorials, letters, and editorials; (2) duplicate data; (3) not a case-control design; and (4) overlapping data or data dumped by the latest reports. Next, we scoured the susceptibility loci identified by GWAS for NCGC in selected articles, 4 lincRNAs that did not overlap with any recognized genes within a 1-MB range of these loci, in either direction (a total span of 2 MB) were eventually identified from the human lincRNAs database [28]. Furthermore, Haploview software 4.2 was used for bioinformatics analysis of haplotype block based on the Chinese Han Beijing (CHB) population data in HapMap (HapMap Data Release 27 Phase II+III, February 2009, on the NCBI B36 assembly, dbSNP b126). These SNPs with minor allele frequencies of greater than 5% in the Chinese population was extracted. There were three haplotype blocks in the Chinese population (**Figure 1A**). The haplotype-tagging SNPs were selected with the Haploview software 4.2 Tagger program; it was found that the rs11752896, rs11752942, rs4714336 and rs4711631 covered the haplotype in block 1 at a 100% frequency (rs11752896 and rs11752942: D′ = 1.0, r^2^ = 1.0; rs11752896 and rs4714336: D′ = 1.0, r^2^ = 1.0; rs11752896 and rs4711631: D′ = 1.0, r^2^ = 1.0). In addition, rs2467950, rs2450764 and rs9312, rs8506 covered the haplotype in block 1 at a 100%, respectively (rs2467950 and rs2450764: D′ = 1.0, r^2^ = 1.0; rs9312 and rs8506: D′ = 1.0, r^2^ = 1.0). SNPs rs2304285 and rs2477757 are outside the blocks. Therefore, the SNPs rs11752896, rs2467950, rs8506, rs2477757 and rs2304285 were chosen as five potential functional SNPs in the exonic of the selected lincRNAs to be analyzed for their associations with risk of gastric cancer.

### Genotyping Analysis

Genome DNA was extracted from peripheral blood lymphocytes of the study subjects. Allele-specific MALDI-TOF mass spectrometry was used to genotype the markers used in the association analyses, as previously described [27], [29]. A total of 60 samples were randomly selected for direct sequencing to confirm the genotyping results from the mass spectrometric analysis, and the results were in 100% agreement. Approximately, 10% of the samples were also randomly selected for a blinded repeat of the genotyping without prior knowledge of the previous genotyping result or the status of being a case and control, and the results were in 100% agreement.

### Cell Culture

The 293T or HGC-27 cells were purchased from the Cell Bank of Type Culture Collection of the Chinese Academy of Sciences, Shanghai Institute of Cell Biology, and were passaged for fewer than 6 months. The 293T or HGC-27 cells were maintained in DMEM with high glucose (Gibco-BRL, Gaithersburg, MD, USA) or RPMI 1640 medium supplemented with 10% heat-inactivated fetal bovine serum (Gibco-BRL, Gaithersburg, MD, USA) and 50 µg/ml streptomycin (Gibco-BRL, Gaithersburg, MD, USA) at a 37°C in the presence of 5% CO_2_.

### Subcellular Fractionation

HGC-27 cells were cultured in a humidified incubator for 2 days. For subcellular fractionation experiments, up to 2×10^6^ cells were used. Cytosolic and nuclear extracts from breast cancer cells were collected using a Nuclear/Cytosol Fractionation kit (Biovision, USA) according to the manufacturer's instructions.

*In-silico* Prediction of Folding Structures Induced by Rs8506G>A in *LincRNA-NR_024015*

As certain structures are more likely to play key roles in biological functions; thus we used RNAfold and SNPfold algorithms to predict the putative influence of rs8506G>A on the local folding structures of *lincRNA-NR_024015* by analyzing the 61-bp regions flanking the polymorphism.

### Construction of Reporter Plasmids

Two reporter plasmids containing 160 bp *lincRNA-NR_024015* exon region fragment flanking the rs8506G or rs8506A allele were synthesized by the Genewiz Company (Suzhou, China) and then cloned into the psiCHECK-2 basic vector (Promega, Madison, WI) (Figure 1B). The resulting construct with *lincRNA-NR_024015* rs8506 SNP (psiCHECK-2-lincRNA-rs8506G and psiCHECK-2-lincRNA- rs8506A) was confirmed by sequencing.

### Transient Transfections and Luciferase Assays

Bioinformatics analysis revealed that the rs8506G>A polymorphism locate at the binding site of microRNA has-miR-526b (http://snpinfo.niehs.nih.gov/). Thereby, the mimics and inhibitors of has-miR-526b (GenePharma Co, Shanghai) were applied to analyze the effect of has-miR-526b on psiCHECK-2-lincRNA-rs8506 reporter genes *in vitro*. The 293T or HGC-27 cells were seeded in 24-well plates (1×10^5^ cells per well) and cultured to 60–70% confluence before transfection; cells were then transfected with the reporter plasmids described above using Lipofectamine 2000 (Invitrogen, CA, USA) as previously described [25]. In each well, co-transfection was performed using 800 ng of constructed plasmid DNA and 0, 1, or 40 pmol microRNA has-miR-526b mimics (Shanghai GenePharma Co., Ltd.), and with or without 40 pmol has-miR-526b inhibitor, according to the manufacturer's instructions. The luciferase activity was measured with the Dual-Luciferase Reporter assay system (Promega, Madison, WI, USA) using a TD-20/20 luminometer (Turner Biosystems, Sunnyvale, CA, USA), and the results were normalized against the activity of the *Renilla* luciferase gene. Each group included 6 replicates, and independent triplicate experiments were performed.

### Expression Vector Construction

To further study the role of *lincRNA-NR_024015* in cancer progression, the full-length cDNA of *lincRNA-NR_024015* harboring rs8506G and rs8506A alleles were synthesized by the Genewiz Company (Suzhou, China) and then cloned into the pcDNA3.1 vectors. The resulting construct with *lincRNA-NR_024015* rs8506 SNP (pcDNA-lincRNA-rs8506G and pcDNA-lincRNA-rs8506A) was confirmed by sequencing.

### RNA Isolation and Quantitative RT-PCR Analysis

Thirty-two NCGC tissue specimens were obtained from biopsies of individual patients and stored at −80°C before analysis. Total RNA was obtained from these cancerous tissues with TRIzol reagent (Molecular Research Center, Inc). cDNA was generated from mRNA using the random primer and Superscript II (Invitrogen) according to the manufacturer's protocol. Real-time quantitative polymerase chain reaction (RT-PCR) was carried out to quantify the relative gene expression of *lincRNA-NR_024015*, using an ABI Prism 7500 sequence detection system (Applied Biosystems) based on the SYBR-green method, and *GAPDH* was used as an internal reference gene in each reaction.

### Cell Visibility Assay

In 96-well, flat-bottomed plates (BD Biosciences, Bedford, MA), 100 µL HGC-27 cells cotransfected with pcDNA-lincRNA-rs8506SNP and has-miR-526b or control were aliquoted into each well. Cell viability was measured by Cell Counting Kit-8 (CCK-8) (Dojindo Laboratory, Kumamoto, Japan) based on the manufacturer's instructions.

### Statistical Analysis

The differences in the distributions of selected demographic variables between cases and controls, as well as the allele and genotype frequencies were assessed by two-sided chi-squared tests. Unconditional logistic regression models were used to estimate the associations of genotypes of SNPs with risk of gastric cancer by odds ratios (OR) and their 95% confidence intervals (CIs), followed by stratification analysis by age, sex, smoking and drinking status. Logistic regression modeling was used in the trend test, as well as to evaluate the potential multiplicative and additive gene-gene and gene-environmental factor interactions. Furthermore, the data were further stratified by sub-groups of the clinic-pathological variables. Statistical power was computed by applying the PS software (http://biostat.mc.vanderbilt.edu/twiki/bin/view/Main/PowerSampleSize, accessed Dec 14, 2010). One-way ANOVA test was used to evaluate the effect of various SNPs on the *lincRNA-NR_024015* transcript expression. All tests were two-sided by using the SAS software (version 9.1; SAS Institute, Cary, NC, USA). A *P*<0.05 was used as the criterion for statistical significance.

## Results

### Genotypes and Risk of Non-cardia Gastric Cancer

In the present study, five SNPs were genotyped between cases and controls. As shown in **Table 2**, a significant association with NCGC risk was observed for rs8506G>A. Specifically, the results of genotyping showed that compared with the rs8506GG or AG genotype, *lincRNA-NR_024015* rs8506AA carrier was significantly associated with risk of non-cardia gastric cancer (adjusted odds ratio [OR] = 1.56, 95%CI = 1.03–2.39). In addition, no significant differences were examined in the other four SNPs (*P*>0.05). Thus, we could conclude that genetic variant rs8506G>A polymorphism in *lincRNA-NR_024015* plays a significantly role in mediating the risk of NCGC.

**Table 2:** Associations between five SNPs in lincRNAs and risk of non-cardia gastric cancer in Chinese population.

Genotype	Patients (438)		Controls (727)		Adjusted OR (95% CI)^a^	*P*_trend_
	No.	(%)	No.	(%)		
**rs11752896**						
AA	222	(50.7)	354	(48.7)	1.00 (Reference)	
AG	183	(41.8)	319	(43.9)	0.90 (0.69–1.16)	
GG	33	(7.5)	54	(7.4)	0.96 (0.61–1.60)	0.25
**rs2467950**						
AA	17	(3.9)	24	(3.3)	1.00 (Reference)	
AG	93	(21.2)	182	(25.0)	0.71 (0.32–1.46)	
GG	328	(74.9)	521	(71.7)	0.88 (0.64–1.75)	0.67
**rs2477757**						
CC	300	(68.5)	516	(71.0)	1.00 (Reference)	
CT	138	(31.5)	211	(29.0)	1.11 (0.84–1.45)	0.37
**rs2304285**						
CC	249	(56.8)	432	(59.4)	1.00 (Reference)	
CT	189	(43.2)	295	(40.6)	1.10 (0.83–1.39)	0.39
**rs8506**						
GG	226	(51.6)	418	(57.5)	1.00 (Reference)	
AG	164	(37.4)	256	(35.2)	1.17 (0.89–1.51)	
AA	48	(11.0)	53	(7.3)	1.66 (1.06–2.60)	**0.01**
GG+AG	390	(89.0)	674	(92.7)	1.00 (Reference)	
AA	48	(11.0)	53	(7.3)	1.56 (1.03–2.39)	

a Data were calculated by logistic regression analysis with adjusted for age, sex, smoking, and drinking status.

### Stratification Analysis of Rs8506G>A Genotypes and Risk of NCGC

We further performed a stratification analysis of the associations between variant genotypes and risk of NCGC by subgroups of clinicopathological features of NCGC in this study. As shown in Table 3, a significant association between the variant genotypes and the risk of NCGC was observed in subjects with smoking (adjusted OR = 2.48, 95%CI = 1.63–3.78, homogeneity test *P* = 0.001), suggesting that smoking modulates the association between the *lincRNA-NR_024015* rs8506G>A variant genotypes and the risk of NCGC. No significant association was found in other subgroups.

**Table 3:** Stratification analysis of the *lincRNA-NR_024015* rs8506G>A genotypes by selected variables in non-cardia gastric cancer patients and controls.

	Cases (N = 438)				Controls (N = 727)				Adjusted OR (95% CI)^a^	*P* value^b^
	GG	(%)	AG+AA	(%)	GG	(%)	AG+AA	(%)	AG+AA vs. GG	
**Age(years)**										
≤60	91	(20.8)	65	(14.8)	194	(26.7)	89	(12.2)	1.56 (1.04–2.34)	
>60	135	(30.8)	147	(33.6)	224	(30.8)	220	(30.3)	1.11 (0.82–1.49)	0.19
**Sex**										
Male	148	(33.8)	174	(39.7)	242	(33.3)	249	(34.2)	1.14 (0.86–1.51)	
Female	78	(17.8)	38	(8.7)	176	(24.2)	60	(8.3)	1.43 (0.88–2.32)	0.44
**Smoking Status**										
Positive	106	(24.2)	83	(19.0)	168	(23.1)	53	(7.3)	2.48 (1.63–3.78)	
Negative	120	(27.4)	129	(29.5)	250	(34.4)	256	(35.2)	1.05 (0.77–1.42)	**0.001**
**Drinking**										
Positive	118	(26.9)	57	(13.0)	186	(25.6)	81	(11.1)	1.11 (0.74–1.67)	
Negative	108	(24.7)	155	(35.4)	232	(31.9)	228	(31.4)	1.46 (1.08–1.98)	0.29
**Family history**										
Positive	15	(3.4)	19	(4.3)	22	(4.0)	29	(4.0)	0.96 (0.40–2.30)	
Negative	211	(48.2)	193	(44.1)	396	(54.5)	280	(38.5)	1.29 (1.01–1.66)	0.52
**H.pylori status**										
H. pylori positive	162	(37.0)	160	(36.5)	418	(57.5)	309	(42.5)	1.37 (1.05–1.78)	
H. pylori negative	64	(14.6)	52	(11.9)	418	(57.5)	309	(42.5)	1.66 (1.12–2.47)	0.42
**TNM stage**										
I	54	(12.3)	50	(11.5)	418	(57.5)	309	(42.5)	1.25 (0.83–1.89)	
II	43	(9.8)	47	(10.7)	418	(57.5)	309	(42.5)	1.48 (0.95–2.29)	
III	86	(19.6)	83	(19.0)	418	(57.5)	309	(42.5)	1.31 (0.93–1.83)	0.71
IV	43	(9.8)	32	(7.3)	418	(57.5)	309	(42.5)	1.01 (0.62–1.63)	

a ORs were adjusted for age, sex, smoking, and drinking status of non-cardia gastric cancer in a logistic regression model.

b *P* value of the test for multiplicative interaction between stratum-related variables and *lincRNA-NR_024015* (rs8506G>A AG+AA *vs.*GG genotypes).

### Cellular Characterization of *LincRNA-NR_024015*

The levels of nuclear control transcript (*U6*), cytoplasmic control transcript (*GAPDH* mRNA), and *lincRNA-NR_024015* were assessed by RT-qPCR in nuclear and cytoplasmic fractions of HGC-27 cells, respectively. The results showed that *GAPDH* mRNA was exclusively detected in the cytoplasmic fraction, while nucleus-retained *U6* was predominantly found in the nuclear fraction. And *lincRNA-NR_024015* expression was predominantly cytoplasmic (**Figure 1B**).

### *In-silico* Analysis of the Effect of Rs8506G>A in *LincRNA-NR_024015* Folding

Using RNAfold and SNPfold algorithms in *in-silico* analysis, we predicted local structural changes of *lincRNA-NR_024015* caused by the rs8506G>A polymorphism located within the exonic region of *lincRNA-NR_024015*. As shown in **Figure 1C and D**, the results suggested that the G to A base change of rs8506G>A directly affects the folding of *lincRNA-NR_024015*, which may affect the binding site for the microRNA. This may then influence the *lincRNA-NR_024015* gene expression.

### Rs8506G>A Genotypes Influence the *LincRNA-NR_024015* Expression by Disrupting the Binding of Has-miR-526b *in vitro*

**Figure 2:**
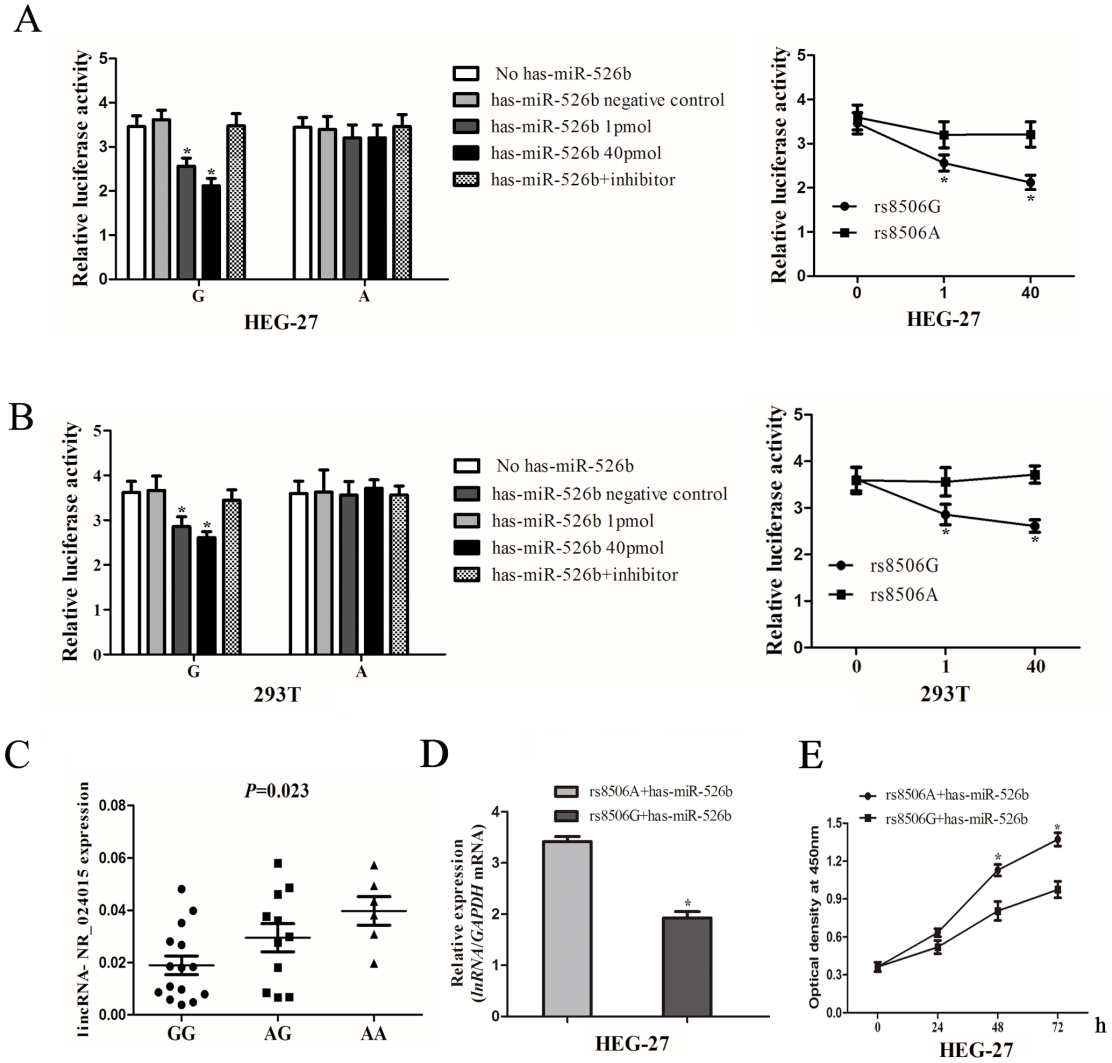
The rs8506G>A genotypes affect *lincRNA-NR_024015* expression. Representative graph of luciferase activity of variant allele on luciferase reporter genes bearing the *lincRNA-NR_024015* exonic region spanning 160 bp flanking the rs8506G>A polymorphism segments in HEG-27 (**A**) and 293T cells (**B**). Results are shown as percentage relative to luciferase activity (Renilla luciferase activity was measured and normalized to firefly luciferase). Relative luciferase activity of the psiCHECK-2-*lincRNA-NR_024015*-G-allele and psiCHECK-2- *lincRNA-NR_024015*-A-allele constructs cotransfected with has-miR-526b mimic and inhibitor. Six replicates for each group and the experiment repeated at least three times. Data are mean ± SEM. Asterisk indicates a significant change (*P*<0.001). (**C**) *LincRNA-NR_024015* expression levels in thirty-two non-cardia gastric cancer patients for different rs8506G>A genotypes (15 rs8506GG, 11 rs8506AG and 6 rs8506AA); data are mean±standard error of the mean. (**D**) HEG-37 cells were cotransfected pcDNA-lincRNA-rs8506G or pcDNA-lincRNA-rs8506A with has-miR-526b. After 24 h, cells were collected, RNA extracted and real time PCR performed. Data are mean±standard error of the mean. “asterisk” represents *P*<0.05. (**E**) Cells' proliferation rate was significantly inhibited when cells cotranfected *lincRNA-NR_024015* haboring rs8506G allele and has-miR-526b. Cell proliferation was performed by the cell viability assay and the effect became obvious from day 2. Six replicates for each group and the experiment repeated at least three times. Data are mean±standard error of the mean. “asterisk” represents *P*<0.05.

Two luciferase reporter gene constructs contained rs8506G or A allele were assayed by transiently co-transfecting with mimic and inhibitor of has-miR-526b that were predicated binding to rs8506G>A polymorphic site by bioinformatics analysis. The result of luciferase activity showed that the HEG-27 cells transiently co-transfected has-miR-526b mimics and construct containing the rs8506G allele exhibited significantly reduced luciferase activity, in a concentration-dependent manner, and the has-miR-526b inhibitors significantly reversed and upregulated their activities. However, no evident change was observed for reporter gene with A allele treated with has-miR-526b mimics or inhibitors (*P*>0.05) (**Figure 2A**). The same results were also observed when these experiments were repeated using 293T cells (**Figure 2B**).

### Association of Rs8506G>A Genotypes with *LincRNA-NR_024015* Expression

We collected 32 tumor tissues from the untreated NCGC patients with different genotypes and performed real-time PCR to evaluate the effects of *lincRNA-NR_024015* rs8506G>A on *lincRNA-NR_024015* expression. The result showed that patients with the rs8506AG and rs8506AA genotypes expressed significantly higher *lincRNA-NR_024015* mRNA levels (mean±SEM) compared to carriers of the rs8506GG genotype (AG: 0.029±0.005; AA: 0.040±0.005; GG: 0.019±0.004; *P* = 0.023), as shown in **Figures 2C**.

### The Effect of MiRNA-dependent Regulation of *LincRNA-NR_024015* Expression on Cell Proliferation

We further investigated whether the *lincRNA-NR_024015* rs8506G>A genotypes have effects on cell proliferation in *vitro*. As showed in **Figure 2D**, *lincRNA-NR_024015* expression decreased after 24 h transfection in cells transiently co-transfected with pcDNA-lincRNA-rs8506G and has-miR-526b compared with those co-transfected with pcDNA-lincRNA-rs8506A and has-miR-526b (*P*<0.001). Cells with decreased expression of *lincRNA-NR_024015* had a weak cell growth rate in comparison with cells transfected with pcDNA-lincRNA-rs8506A and has-miR-526b from day 2 (*P* = 0.004) (**Figure 2E**).

## Dicussion

In the present hospital-based case-control study containing a total of 438 patients and 727 healthy controls, our group found the rs8506G>A is associated with risks of NCGC. Our data showed that subjects carrying the rs8506AG and rs8506AA genotypes had a significant increased risk for NCGC compared with the GG genotype (*P*<0.05). Additionally, it appeared that a high risk effect of this polymorphism was more pronounced in smoking subjects. To our knowledge, this is the first study to comprehensively evaluate the association between the variants in exonic of lincRNA and risk of NCGC.

As another class of regulatory noncoding RNAs, lincRNAs, have recently moved to the forefront of noncoding RNA study. These mRNA-like molecules, lack a significant open reading frame, are generally capped, spliced and polyadenylated have been implicated in a wide range of cellular processes such as nuclear architecture, regulation of gene expression, immune surveillance, or embryonic stem cell pluripotency. A handful of studies have demonstrated lincRNAs emerged as a new aspect of biology in a variety of disease states, and changes in expression levels of lincRNAs may contribute to cancer biology at transcriptional, post-transcriptional and epigenetic levels [18], [30]–[32]. One prominent lincRNA, *ANRIL*, had been functionally implicated in cancer progression, typically repressing epigenetic gene expression via binding to and recruiting chromatin modifying complexes [33], [34]. Another famous example is the lincRNA, *GAS5*; genetic aberrations at this lincRNA locus have been found in many types of tumors, including melanoma, breast, and prostate cancers [35]–[37]. These lines of evidence supported the importance of lincRNAs in cellular biology and oncogenesis. Recently, two GWASs have reported a subset of NCGC susceptibility loci (5p13.1, 3q13.31, 8q24.3, 6p21.1 and 7p15.3) that associated with the development of NCGC. Bioinformatics analysis revealed several lincRNAs closed to these loci. Furthermore, several relevant single nucleotide polymorphisms (SNPs) located in the exonic regions of lincRNAs that may associate with NCGC were identified. As we all known, over the past decade, with the availability of large-scale RNA sequencing, it is now becoming remarkably clear that thousands of disease-related genetic variants resided outside of genes or even in non-coding transcripts have already been obtained by genome project in mammals [24]. Recent emerging evidences have indicated the association between SNPs resided in lincRNAs and human cancers. For example, based on the 1000 Genomes data, Guangfu Jin and colleages have found that regions of lncRNA had a SNPs density similar to protein-coding regions and further annotated the phenotype-related SNPs reported by GWAS at lncRNA region may contribute to prostate risk [38]. One recent study also reported that a genetic polymorphism in *lincRNA-uc003opf.1*gene is associated with an increased risk of developing esophageal squamous cell carcinoma in Chinese populations [39]. Collectively, our present study is consistent with previous findings showed that *lincRNA-NR_024015* was moderately more abundant in the cytoplasm than in the nucleus of fractionated gastric cancer cells, suggesting that the function of this lincRNAs is exerted in the cytoplasm. Our results provided a strong evidence supporting a hypothesis for cytoplasmic regulation, in which the *lincRNA-NR_024015* rs8506G>A SNP may affect the expression of this lincRNA by modifying the binding site for the has-miR-526b.

In the present study, our result of association between a genetic polymorphism in the exonic regions of a lincRNA and susceptibility to NCGC was firstly obtained in Chinese populations. The relatively large sample sizes used decreased the size of the ORs that can be detected statistically. Moreover, we have achieved a study power of over 90% (two-sided test, α = 0.05) in detecting an OR of 1.28 for the rs8506AG+AA genotypes (occurring at a frequency of 42.5% amongst the controls), when compared with the rs8506GG genotype. Notably, the association is biologically plausible and is consistent with the findings of our functional studies.

In conclusion, the present study provided the first evidence that genetic polymorphisms in the exonic regions of lincRNAs play a vital role in mediating individual susceptibility to NCGC. Our results further support the hypothesis that genetic variants in lincRNA exonic regions may affect microRNA-mediated regulation and associated with the risk of GC. Our findings warrant validation in larger, preferably population-based, case-control studies, as well as by well-designed mechanistic studies.
